# Density based pruning for identification of differentially expressed genes from microarray data

**DOI:** 10.1186/1471-2164-11-S2-S3

**Published:** 2010-11-02

**Authors:** Jianjun Hu, Jia Xu

**Affiliations:** 1 Department of Computer Science and Engineering, University of South Carolina, Columbia, SC, 29208, USA

## Abstract

**Motivation:**

Identification of differentially expressed genes from microarray datasets is one of the most important analyses for microarray data mining. Popular algorithms such as statistical t-test rank genes based on a single statistics. The false positive rate of these methods can be improved by considering other features of differentially expressed genes.

**Results:**

We proposed a pattern recognition strategy for identifying differentially expressed genes. Genes are mapped to a two dimension feature space composed of average difference of gene expression and average expression levels. A density based pruning algorithm (DB Pruning) is developed to screen out potential differentially expressed genes usually located in the sparse boundary region. Biases of popular algorithms for identifying differentially expressed genes are visually characterized. Experiments on 17 datasets from Gene Omnibus Database (GEO) with experimentally verified differentially expressed genes showed that DB pruning can significantly improve the prediction accuracy of popular identification algorithms such as t-test, rank product, and fold change.

**Conclusions:**

Density based pruning of non-differentially expressed genes is an effective method for enhancing statistical testing based algorithms for identifying differentially expressed genes. It improves t-test, rank product, and fold change by 11% to 50% in the numbers of identified true differentially expressed genes. The source code of DB pruning is freely available on our website http://mleg.cse.sc.edu/degprune

## Introduction

Statistical methods for identifying differentially expressed genes are now routinely used by biologists. There are two main categories of algorithms. The first category includes single gene testing approaches such as fold change [1], rank product [2], t-test and its variants [3]. These methods are characterized by a single statistics score used to rank genes from significantly differentially expressed genes to no- change ones. The second category includes gene set testing approaches such as gene set enrichment analysis [4,5]. These methods are featured by exploiting externally determined gene sets to rank a group of genes. The shortcoming of these methods is that in many cases such gene set information is not available, especially for under-studied species. Despite increasing usage of gene set analysis methods [4], single-gene based identification algorithms for differentially expressed genes (DEGs) still dominate the practice of biological differential gene expression analysis [6-9] from microarray data. This is partially due to their simplicity as well as little requirement on gene annotation. Thus improving single-gene DEG identification algorithms still has great implication for DEG microarray analysis practice in biology. Currently, a major purpose of DEG algorithm design is to reduce their false positive and false negative errors since experimental biologists usually only afford to test only a very limited number of predicted DEGs.

Biologically interesting DEGs are those genes that have significant phenotypic changes along with their change of gene expression levels. Most current single-gene DEG identification algorithms, however, take it as a statistical significance test problem without referring to the real characteristics of differentially expressed genes [10]. Unfortunately, limited number of samples of microarray datasets in most biological studies makes such statistical test methods ineffective [11,12]. This issue has been addressed recently using multiple strategies. A popular strategy is to gather information across similar genes to improve DEG identification. This includes the Bayes t-test approach [13], the local pooled error algorithm [14], the famous SAM algorithm [15], and et cetera. Another strategy is to use external information to improve variance estimation. Wille et. al. [16] proposed an external variance estimation algorithm called EVE, which exploits the relationships between variances of gene expression and gene function. Kim and Park [17] proposed a normalization method to make multiple microarray datasets with different chips comparable, which then facilitates the estimation of gene variance with those external datasets. Their method showed big improvement over the basic regularized t-test algorithm on experiments with 1x1, 2x2 and 3x3 samples. Hack- stadt and Hess [18] investigated three filtering methods for pruning genes before statistical tests: MAS detection call, variance, and average signal. They showed that gene filtering by MAS detection call and mean signal lead to increased performance of DEG identification. They also suggested that filtering 50% of probe sets is reasonable due to majority genes are expected to be equally expressed.

**Figure 1:**
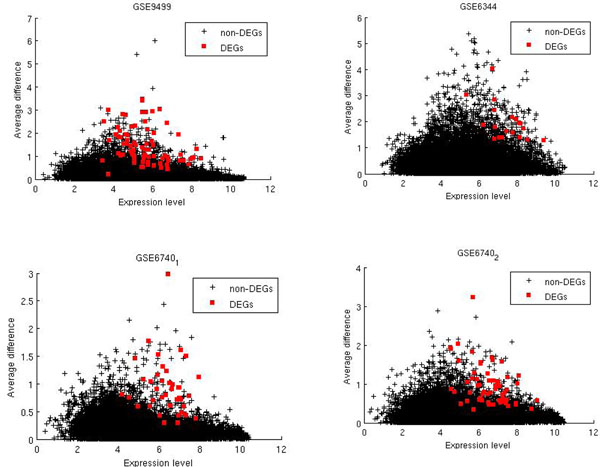
Distribution of true DEGs in the boundary regions of the AG-AD feature space for four datasets. Most DEGs are located in the boundary regions in the figure. Screening out boundary genes has the potential to improve the power of gene ranking methods such as t-test for DEG identification.

Here we propose a pattern recognition strategy for improving DEG identification algorithms. This pruning algorithm shares some similarity to all-gene-analysis DEG identification algorithms as discussed in [19]. The first step of our algorithm is to apply pattern recognition algorithms to prune non-DEGs from the whole gene list based on the characteristics of experimentally verified differentially expressed genes. In this paper, a density based pruning (DB Pruning) is developed based on two features of DEGs: the average difference of gene expression level between two classes and the average gene expression level. It is motivated by the observation that a majority of true DEGs experimentally verified by RT-PCR tend to have high expression levels in 38 real-world datasets [20]. The tendency for high expression genes to be over-represented in the list of DEGs is also suggested in [18]. And we found that the true DEGs tend to be sparsely located in the boundary regions in the average-difference—expression level space (peripheral areas of the gene distribution map) as shown in Fig. 1. In the second step, common statistics-based DEG identification algorithms such as t-test are applied to rank genes. The non-DEG pruning will be able to enrich true DEGs in the remaining gene lists. This is especially desirable for small-size microarray datasets. It can also be used to greatly reduce the computational cost of DEG algorithms that search gene combinations [21], in which all gene-pairs need to be ranked. Based on systematic evaluation on 17 real-world microarray datasets with a total of 184 true DEGs applied to four existing DEG algorithms, we showed that DB pruning can significantly improve the performance of these traditional algorithms for both large and small datasets in terms of false positive rates. For example, DB Pruning can prune 83% (19156) genes out of 22283 while keeping 89% (164) true DEGs out of 184 for 17 datasets we tested. The enrichment of true DEGs in the pruned gene list is almost six times of the original gene list. DB pruning is also shown to increase the AUC score of rank product by up to 21% and helps it to find 33% more true DEGs when the cutoff top K=550. For fold change and t-test, it can find 11% and 15.8% more true DEGs. When the sample size is 4x4, DB pruning improves t-test by 26.8% in terms of the number of identified true DEGs. When the sample size is 2x2, t-test is improved by 50%.

## Methods

Differentially expressed genes are those genes with significant difference in expression levels among two or more classes/conditions. Such expression changes should not be caused by random variation in gene expression. DEGs resulting from a specific perturbation to corresponding pathways tend to share some functional or physiological characteristics. It is thus justified that DEG identification can be improved by considering the characteristics of true DEGs. One characteristic is that true DEGs experimentally identified to date tend to have high average expression values across all conditions [20]. The resulting WAD algorithm is very competitive compared to other DEG algorithms based on evaluation over 38 GEO microarray datasets. In WAD, the product of the gene expression ratio and relative gene expression level are used to rank genes. The limitation of this ranking scheme is that it biases to genes with balanced expression ratios and expression levels.

The main idea of the proposed DB pruning is that differentially expressed genes between two conditions are usually located in the boundary region in the 2-D feature space of average gene expression (AG) versus average difference of gene expression (AD). Fig.1. shows the distribution of true DEGs in the 2D space for four datasets: GSE9499, GSE6342, GSE6740_1, and GSE6740_2 from GEO database [22]. Based on the fact that boundary region is characterized with scarcity of genes, a density based pruning algorithm is proposed here for pre-filtering non-DEG genes located outside the boundary region so that the false positive rate of current DEG algorithms can be improved.

### Density-based pruning algorithm for DEG identification

The main idea of density based pruning is to remove non-DEGs that usually appear within the dense part of the AG-AD space. Assume M is a microarray matrix with N genes (rows) and P profiles (columns). There are total P1 profiles in P corresponding to condition A and P2=P-P1 profiles corresponding to condition B. The average expression level (AG) of a gene *X_i_* is defined as (*X_i_^A^* + *X_i_^B^*)/2, where *X_i_^A^* and *X_i_^B^* are the average expression level (log-scaled) of gene *X_i_* under condition A and B. The average difference of gene expression of a gene *X_i_* is defined as |*X_i_^A^* - *X_i_^B^|* Since the expression values used in calculating |*X_i_^A^* - *X_i_^B^|* are log-transformed, the average differences of expression calculated here are actually equivalent to expression ratios as in fold change method.

The density based pruning algorithm works as follows: each gene *X_i_* is mapped into the (AG, AD) feature space. Pairwise Euclidian difference between two genes *X_i_* and *X_i_*is calculated, where *i* ≠ *j*. If the distance is smaller than a user-defined radius threshold *R_0_*, these two genes will be declared as neighbors. Then the number of neighbors (*n _i_*) will be calculated for each gene. If *n _i_* ≥ *N*_0_ then gene *X_i_* will be pruned from the gene list, where *N*_0_ is a user-specified density parameter. The final output gene list is composed of genes that are mostly outliers located in the boundary region of the AG-AD feature space.

For different datasets, an important step of our algorithm is to determine appropriate parameters *R*_0_ and *N*_0_ such that all or most DEGs are kept in the final list and that a maximum number of non-DEGs are pruned. Through our experiments, we found that *N _n_* = 4 is an appropriate parameter for most of 17 datasets used in our experiments. The value of threshold radius *R*_0_ has a large effect on the number of pruned genes. The minimum value of *R*_0_ is 0 and maximum value is the max AD value. A binary search procedure is used to identify *R*_0_ value that can generate the desired number (K) of candidate DEGs.

### DEG identification algorithms

We tested four popular DEG identification algorithms on the 17 GEO datasets with or without DB pruning.

• Fold Change (FC) is one of early DEG identification algorithms that are still widely used by biologists. It was recently recommended to be used with a non-stringent P cutoff to generate more reproducible DEG lists [11,23]. FC ranks genes based on the ratio of average gene expression under two conditions. Usually a 2-fold change is regarded as significant in many biological studies. A major criticism of FC is that it doesn’t consider the case that genes with low expression level in both conditions but with small variances can be ranked high.

• Rank Product (RP) [2,24] ranks genes based on product of rank ratios for multiple A-B conditions. The results and simplicity of RP is similar to FC but overcomes its most significant limitations. It also provides a statistically rigorous estimation of significance. It was reported to have good performance for small or noisy datasets.

• T-statistics (tTest) is one of the earliest and popular methods used in DEG identification. The major advantage is that it considers the variation of genes in its ranking. The limitation is that the estimation of gene expression variances is not reliable for small datasets, which can lead to poor performance.

• Weighted Average Difference (WAD) [20] is a DEG algorithm based on the observation that experimentally verified true DEGs tend to have high expression level across the conditions. Genes are ranked by the product of fold change times normalized expression level. It was shown to have significantly better and robust performance than most other standard algorithms including FC, RP and tTest.

For each of these methods, we compare their DEG prediction performance with or without DB pruning. Comprehensive evaluation is conducted on 17 real-world microarray datasets from GEO database with experimentally verified DEGs.

## Results

### Data set preparation

Most DEG identification algorithms are tested using unverified “statistically” significant genes plus a few (if any) experimental verifications [2,3,16,25,26]. These unverified DEGs may be quite different from biologically meaningful DEGs and have bias toward statistical algorithms. In this paper, we used real-world microarray datasets with experimentally verified DEGs as collected by Kadota et. al. [20]. They collected 38 microarray datasets with experimentally determined true DEGS by real-time polymerase chain reaction (RT-PCR). Thirty six of the datasets are downloaded from GEO database [22]. Without losing generality, we experimented with 17 disease or dose response datasets of Homo sapiens out of the 36 GEO datasets (Table 1). The 17 datasets are reported just for convenience so that we can use a single set of DB pruning parameters. Other datasets have also been tested with different DB pruning parameters and similar results are obtained. All 17 datasets are normalized and transformed into log scale. Table 1 shows the statistics of the datasets with the sample sizes of normal and disease conditions and also the number of true DEGs. Out of the 17 datasets, only 7 have more than 10 samples for both conditions. Four datasets have less than 5 samples per condition. These datasets show that real-world GEO datasets, especially historical ones, tend to have small sample size.

**Table 1:** 17 Datasets with 284 DEGs in total. Each dataset has 22833 genes.

Dataset	Conditions		True DEG

	A	B	
GSE1462	4	4	4

GSE1615_1	4	5	8

GSE1650	18	12	8

GSE2666_2	5	5	6

GSE3524	16	4	4

GSE3860	9	9	8

GSE4917	3	3	5

GSE5667_1	5	6	3

GSE6236	14	14	7

GSE6344	10	10	19

GSE6740_1	10	10	40

GSE6740_2	10	10	62

GSE7146	6	6	6

GSE7765	3	3	13

GSE8441	11	11	9

GSE9499	15	7	77

GSE9574	15	14	5

The 17 Datasets used here cover a variety of biological or medical studies: GSE1462 (mitochondrial DNA mutations), GSE1615_1 (Valproic acid treatment), GSE1650 (chronic obstructive pulmonary disease), GSE2666_2(bone marrow Rho level effect), GSE3524 (tumor of epithelial tissue), GSE3860 (Hutchinson-Gilford progeria syndrome), GSE4917 (breast cancer), GSE5667_1 (atopic dermatitis), GSE6236 (Adult vs. fetal reticulocyte transcriptome comparison), GSE6344 (renal cell carcinoma disease), GSE6740_1 (HIV-infection), GSE6740_2 (HIV-infection, disease state), GSE7146 (hyperinsulinaemic, does response), GSE7765 (dose response, DMSO or 100 nM Dioxin), GSE8441 (dietary intake response), GSE9574 (breast cancer), and GSE9499 (hypomorphic germline mutations). The diversity of these datasets ensures that the observed performance of the proposed pruning algorithm is not due to some specific characteristics of the data.

### Bias of DEG identification algorithms

DEG identification algorithms such as t-test and fold change all have different bias in their ranking schemes. Three factors have been commonly used in their gene ranking criteria: *r*(*y*) = (*d, e, v*) where *d* is the difference of expression levels between two conditions; *v* is the overall gene expression level of the gene; and *v* is the variance of the gene’s gene expression. T-statistics based algorithms may make false positive prediction for genes with low *d* because of small *v***.** Fold change algorithm instead suffers from the fact that a gene with large variances tend to have larger fold changes. Both methods may make mistakes by neglecting the overall gene expression levels, which has been explicitly addressed by the WAD algorithm which rank genes by *d* × *e*. Indeed, it is shown that when the expression level was considered, the WAD algorithm achieves significantly better prediction performance than all previous methods based on extensive tests on 38 datasets with known true DEGs. This shows that expression levels of true DEGs are usually high. It is thus interesting to visualize the bias of different DEG algorithms in the (*d, e*) feature space. For simplicity, the variance***v*** feature is neglected as it is not correlated to true DEGs as strongly as (*d, e*) features.

**Figure 2:**
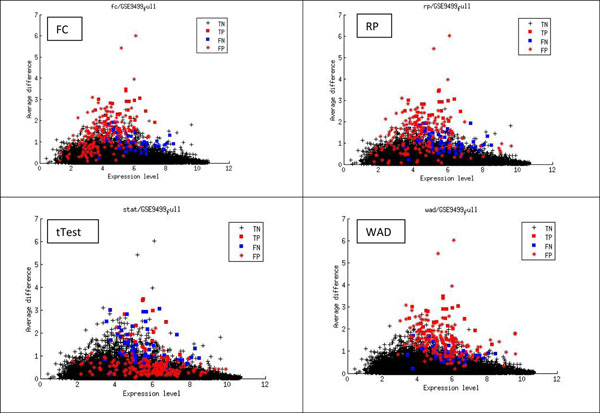
Visualization of bias of popular DEG identification algorithms. FC has many false positive predictions for genes with low average expressions or small expression differences. RP’s false positives are sparsely located in low expression and small average difference region. tTest’s false positives are dominated by genes with low average difference. WAD has less false positives than other algorithms.

In Fig. 1, it is shown that most true DEGs are outliers located in the sparse boundary region in the (*d, e*) space. A smaller portion of true DEGs are mixed with other non-DEGs in the dense regions and cannot be differentiated by the algorithms such as WAD and FC. To illustrate the bias of popular DEG identification algorithms, Fig. 2 shows true positive (TP), true negative (TN), false positive (FP), and false negative (FN) DEGs for dataset GSE9499 which has 77 true DEGs. Fig.2. (a) shows that fold change (FC) misses most true DEGs (FN genes), which are located in the region below the threshold average difference and with high expression levels. This is because FC uses a fixed ratio as cutoff value. It also made many false positive predictions mostly in the region with low expression levels (see FP genes). Rank product (Fig.2b) misses similar true DEGs as fold change algorithm does but the false positive genes have different distributions. Fig. 2 (c) shows the predicted DEGs of t-test. This method misses many true DEGs that have high average difference between two conditions. Most of its false positives are located across the expression level with low average difference, reflecting the fact that it can be misled by genes with small variances. Fig.2 (d) shows the distribution of predicted DEGs of WAD algorithm. WAD has a better performance in terms of capturing true DEGs located in the boundary region. The main false positives are mixed together with the true DEGs, which are difficult to distinguish without extra information.

### Improving DEG identification algorithms using density based pruning

#### Effect of density based pruning of non-DEGs

One way to test the performance of DB pruning is to calculate the enrichment score of true DEGs after pruning. It is defined as the ratio of true DEGs and the number of all genes. We applied DB pruning algorithm to 17 microarray datasets each having 22283 genes. For simplicity, we use the same set of parameters (*N*_0_ = 4 and *R*_0_ = 0.0017) to prune all the datasets. The idea is that by pruning those non-DEGs, true DEGs are more enriched in the remaining gene list and should be easier to be identified by current DEG algorithms. The reduced search range of candidate DEGs can also greatly reduce computational cost for detecting combinatorial gene sets. Table 2 shows the effect of applying DB pruning. It shows that this procedure can prune more than ≥86% (19156) genes out of 22283 while keeping ≥89% (164) out of 184 true DEGs. The enrichment of true DEGs in the pruned gene sets is 6 times of original gene list. Note that a binary search procedure is available to determine the DB pruning parameters for obtaining a user-specified number of candidate DEGs.

**Table 2:** Comparison of No. of missing true DEGs after DB pruning. (*N*_0_ = 4, *R*_0_ = 0.0017)

Total Gene: 22283	After DP-pruning	True DEG	DP missed
GSE1462	2054	4	0

GSE1615_1	2449	8	3

GSE1650	1317	8	2

GSE2666_2	1618	6	2

GSE3524	814	4	0

GSE3860	2073	8	0

GSE4917	785	5	1

GSE5667_1	1316	3	0

GSE6236	2231	7	0

GSE6344	3127	19	0

GSE6740_1	1183	40	1

GSE6740_2	1801	62	5

GSE7146	1274	6	1

GSE7765	1607	13	1

GSE8441	978	9	1

GSE9499	1805	77	3

GSE9574	1448	5	0

#### Improvement of ranks of true DEGs in the rank list by DB pruning

Here we check how DB pruning can improve existing DEG identification algorithms. We compare the ranks of true DEGs in the original gene list and in the pruned gene list ranked by different algorithms including t-test, fold change, rank product and Wad. Table 3 shows that after DB pruning, ranks of most true DEGs by t-test and fold change statistics are improved, usually with significant improvements. For example, ranks of true DEG were improved from 1404 to 808, 3800 to 1713, 1321 to 768 and etc by the t-test after DB pruning. We also observe considerate improvements of DEG ranks for fold change and rank product algorithms via DB pruning. The improved enrichment of true DEGs toward top of gene rank lists implies that it can help current DEG algorithms to achieve better performance as shown in next subsections. DB pruning has moderate effect on Wad algorithm since Wad also used the feature of true DEGs, namely, the tendency of true DEG to have high expression levels and high expression difference.

**Table 3:** Ranks of true DEGs in original gene list and pruned gene list. Genes are sorted by four DEG identification algorithms on the GSE1577 dataset. Increase of ranks of true DEGs means that DB pruning have correctly filtered out many non-DEGs.

t-test/tTest’	1404/808	7/6	1321/768	3800/1713	4741/1975	3633/1659	4145/1828	606/388	210/155
FC/FC’	167/153	154/142	39/33	18/13	1/1	22/17	6/5	1601/1249	80/72

Rp/Rp’	111/85	91/70	18/12	9/8	1/1	16/15	6/6	4520/980	97/68

Wad/Wad’	31/31	25/25	32/25	7/7	3/3	15/15	6/6	515/515	10/10

#### Improving standard DEG algorithms using DB pruning

To evaluate the improvement of prediction performance of DEG identification algorithms with DB pruning, we used the receiver operating characteristic (ROC), or simply ROC curve. It is a graphical plot of the fraction of true positives (TPR = true positive rate) vs. the fraction of false positives (FPR = false positive rate) as the K (the number of genes predicted to be DEGs) varies. We use the area under curve (AUC) value of the ROC curve as the criterion for comparison, which has been used in previous work [20]. To make the comparison relevant to real-world practice, we only plot and compare the AUC value with K varies from 1 to 1000 rather than to the total number of genes (22832) as done previously. The reason is that biologists rarely have the resources to check all 22832 genes and usually only care about top K predicted DEGs for experimental verification.

We calculate AUC values with K up to 1000 for four DEG algorithms with or without DB pruning. The experiments are conducted on all 17 datasets with a total of 284 true DEGs. DB priming is run with the following parameters: neighborhood radius *R*_0_ = 0.03, no. of neighbors, *N*_0_ = 4. Table 4 shows that DB pruning significantly increased the AUC values for all four popular DEG algorithms especially for rank product algorithm with 21% increase of AUC score. The smallest improvement is for WAD algorithm, which is reasonable considering that WAD uses the same information as DB pruning, though in different way. To obtain more intuitive understanding of how DB pruning improves current DEG algorithms, we showed in Table 5the total numbers of true DEGs out of top K predictions identified by different algorithms from the 17 datasets with or without DB pruning. First, the results showed that rank product and fold change have worse performance than tTest and WAD algorithms in the number of identified true DEGs. For example, tTest and WAD can detect 132 and 156 true DEGs from the 17 datasets when K=150 predictions are allowed for each dataset. Instead, RP and FC can only detect 74 and 97 true DEGs respectively. When the no. of predictions K increases, all algorithms retrieve more true DEGs with the highest coverage by WAD algorithm which retrieves 240 out of 284 true DEGs when 550 genes are allowed to predict for each dataset. A major observation of Table 4 is that all 4 algorithms can benefit from DB pruning with the maximum improvement for tTest and the minimum improvement for WAD. When DB pruning is used, the 38 true DEG discrepancy between tTest and WAD is reduced to 6 when K=550. In other words, DB pruning can significantly improve the performance of t-statistics based DEG identification algorithms up to level of the most competitive DEG algorithm-WAD. In the case of RP, DB pruning helps RP to find 35 (nearly 33%) more true DEGs for K=550. For FC and tTest, 11% and 15.8% more true DEGs are identified with the help of DB pruning. The DB pruning shows only moderate to zero improvement for WAD algorithm because they use the same information, the gene expression levels and average difference of gene expressions.

**Table 4:** Increase of AUC values for DEG algorithms after DB pruning: Rp, Wad, Fc, and tTest.

	Partial AUC (up to K=1000)	Percentage of Improvement
Rp/Rp’	0.0162/0.0196	21%

Fc/Fc’	0.0245/0.0263	7.3%

tTest/tTest’	0.0284/0.0310	9.2%

Wad/Wad’	0.032/0.033	3.1%

**Table 5:** Increase of No. of identified true DEGs out of top K predictions with or without DB pruning. Rp’, Wad’, tTest’, FC’ are algorithms with DB pruning. The total number of true DEGs of the 17 datasets is 284.

	K=150	K=250	K=350	K=450	K=550
Rp/Rp’	74/78	81/91	92/104	98/122	106/141

Fc/Fc’	97/98	120/137	146/159	164/184	178/198

tTest/tTest’	132/150	163/181	179/206	191/218	202/234

Wad/Wad’	156/156	195/198	221/221	227/227	240/240

**Figure 3:**
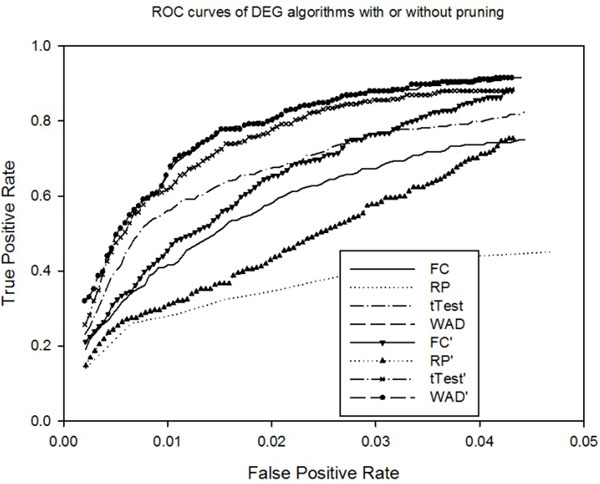
Comparison of ROC curves of DEG algorithms with/out DB pruning. It shows that WAD and t-Test have higher AUC values than FC and RP. Using DB pruning, tTest’s AUC value can be improved to be close to that of WAD. Actually, DB pruning significantly improves all DEG algorithms.

To further investigate the improvement of DB pruning over classic DEG algorithms, Fig. 3. shows the ROC curves of the algorithms with and without pruning with K=1 to 1000. It is shown that WAD algorithm has the best performance and there exists dominance relationship of WAD>t-test>FC>RP. It also shows that after DB pruning, the performance of t-test was significantly improved up to that of WAD algorithm. Both RP and FC were also greatly improved. Indeed, Fig.3 clearly demonstrates that DB pruning is able to significantly improve the AUC values with improvements across all K values ranging from 1 to 1000. Compared to the improvements of ROC curves as shown by the variance estimation algorithms [22], our improvement is much more significant.

#### DB Pruning’s performance on microarray datasets with a small number of samples

A major issue of current DEG algorithms is that they have difficulty to deal with small-size microarray datasets. Unfortunately, many biological studies only generate limited profiling samples due to cost or labor constraints. Here we show DB pruning can help improve the performances of popular DEG algorithms for such small datasets. Four algorithms were applied to 17 datasets with a total of 284 true DEG. These full datasets are sub-sampled to generate small datasets with 2, 3, and 4 samples for each condition. Each algorithm was also run with different K, the number of predicted DEGs by DEG algorithms. Table 6 shows how the number of samples of the data set affects the number of true DEGs predicted by current algorithms with or without DB pruning.

**Table 6:** The no. of predicted true DEGs using partial samples from condition A and B with or without using DB Pruning. Rp’, Wad’, tTest’, FC’ are algorithms with DB pruning. The total number of true DEGs of the 17 datasets is 284.

		K=150	K=250	K=350	K=450	K=550
2x2 samples	Rp/Rp’	45/46	61/61	68/71	78/79	86/92

	Fc/Fc’	43/44	58/62	62/71	69/82	74/89

	tTest/tTest’	16/32	24/47	31/60	37/77	45/88

	Wad/Wad’	92/92	116/116	127/128	140/141	149/149

3x3 samples	Rp/Rp’	52/53	60/64	71/75	76/81	81/90

	Fc/Fc’	54/54	61/63	71/77	79/87	90/100

	tTest/tTest’	32/52	48/76	62/98	72/115	82/132

	Wad/Wad’	91/91	128/129	150/150	166/166	171/175

4x4 samples	Rp/Rp’	60/63	70/74	78/86	85/97	97/105

	Fc/Fc’	62/63	74/81	86/92	94/108	115/128

	tTest/tTest’	67/85	83/111	100/141	108/157	119/174

	Wad/Wad’	119/119	155/155	173/174	189/190	193/196

Firstly, the table shows that with the increase of K, more true DEGs will be predicted. For each specific K, decreasing the number of samples reduces the number of true DEG identified. For example, when the number of samples of each condition decreases from 4 to 2, the number of predicted true DEGs will drop from 119 to 92 for WAD, from 60 to 45 for RP, from 62 to 43 for FC, and from 67 to 16 for tTest, which has the largest reduction of performance. DB pruning is shown to be able to significantly improve the prediction performance, especially for tTest, RP and FC. With 4x4 samples, DB pruning helps tTest to identify 18 more true DEGs, a 26.8% improvement. When the sample size is reduced to 2x2, the improvement is 16, or a 50% improvement. Improvement upon RP and FC is less significant, but still achieves 20% improvement when K=550 for FC with 2x2 samples and 14% for RP with K=450 and 3x3 samples. All these prove that the DB pruning is a useful procedure for DEG identification.

## Discussion

We have proposed a density based pruning algorithm for removing non-differentially expressed genes with high confidence from the total gene list. This pruning procedure can significantly improve the prediction accuracy of popular DEG identification algorithms such as fold change, t-test, and rank product. The key idea of DB pruning is based on the observation that DEGs tend to have high average expression values across conditions.

In this paper, the golden standard true differentially expressed genes are those verified by the RT-PCR method, which may comprise of only a portion of true DEGs. The fact that most true DEGs used here show high average expression levels may be due to the technical limitation of RT-PCR and/or microarray: only highly expressed genes can be identified. In this case our method should be qualified to be able to improve DEG identification algorithms for these types of true DEGs.

DB pruning has two parameters to set to pre-filter non-DEGs. Even though there is no theoretical guideline for setting their perfect parameter values, these two values can be easily set to achieve significant improvements. Both parameters can be set such that an expected number of predicted DEGs are obtained. In our experiments, a single set of R0 (=0.0017) and N0 (=4) have been able to reduce the DEG prediction accuracy for all 17 datasets. This demonstrates the stability of the algorithm in terms of the parameters for different datasets. In addition, an improved pruning algorithm based on Pareto set concept is being developed which can completely remove the parameters in DB pruning.

There are several further improvements following this pattern recognition based DEG identification. One common problem of DEG identification is lack of sufficient data points for reliable estimation of gene expression levels and their differences. This usually hurt the performance of most DEG algorithms including our pruning algorithm. One potential is to use additional external datasets to help estimate gene expression levels and their differences for the dataset of the study. Our preliminary experiments showed that estimating gene expression levels using external datasets is straightforward and feasible but estimating difference of gene expression needs more study. Another improvement is to introduce additional features of DEGs, e.g. the variance of gene expressions across multiple datasets. For example, the variance estimation method using multiple datasets [16] can be combined with DB pruning algorithm. Functional annotation information from gene ontology or pathways can also be integrated to aid gene pruning. Current DB pruning focuses on identifying DEGs between two groups. The extension to multiple groups is straightforward since calculation of average expression level remains the same. And the average difference of expression can be defined as sum of average difference among pairwise comparisons.

Our DB pruning is implemented using C++ and Perl and can be downloaded from http://mleg.cse.sc.edu/degprune.

## Authors’ contributions

JH initiated the project, proposed the DB pruning idea, helped in experimental designs, and wrote the manuscript. J. X. developed the code, conducted the experiments, and collected the data. Both authors read and approved the manuscript.

## Competing interests

The authors declare that they have no competing interests.
